# ﻿Cytotaxonomic investigations on species of genus *Narcissus* (Amaryllidaceae) from Algeria

**DOI:** 10.3897/compcytogen.v16.i1.78852

**Published:** 2022-04-05

**Authors:** Naila Chahinez Boukhebache, Nabila Amirouche, Rachid Amirouche

**Affiliations:** 1 University of Sciences and Technology Houari Boumediene, Faculty of Biological Sciences, Lab. LBPO, Team Biosystematics, Genetic and Evolution, USTHB, PO Box 32, El-Alia, Bab-Ezzouar, 16111 Algiers, Algeria University of Sciences and Technology Houari Boumediene Algiers Algeria

**Keywords:** Amaryllidaceae, chromosomes, karyotype, *Narcissus*, North-Africa, polyploidy

## Abstract

This paper provides new cytotaxonomic data on the genus *Narcissus* Linnaeus, 1753, in Algeria. Populations of seven taxa, *N.tazetta* Linnaeus, 1753, *N.pachybolbus* Durieu, 1847, *N.papyraceus* Ker Gawler, 1806, *N.elegans* (Haworth) Spach, 1846, *N.serotinus* sensu lato Linnaeus, 1753, including *N.obsoletus* (Haworth) Steudel, 1841, and *N.cantabricus* De Candolle, 1815, were karyologically investigated through chromosome counting and karyotype parameters. *N.tazetta* and *N.elegans* have the same number of chromosomes 2*n* = 2*x* = 20 with different karyotype formulas. Karyological and morphological characteristics, confirm the specific status of *N.pachybolbus* and *N.papyraceus*, both are diploids with 2*n* = 22 but differing in asymmetry indices. The morphotypes corresponding to *N.serotinus* sensu lato show two ploidy levels 2*n* = 4*x* = 20 and 2*n* = 6*x* = 30 characterized by a yellow corona. Some hexaploid cytotypes have more asymmetric karyotype with predominance of subtelocentric chromosomes. They are distinguished by orange corona and may correspond to *N.obsoletus*. Other cytotype 2*n* = 28 of *N.serotinus* was observed in the North Western biogeographic sectors. *N.cantabricus* was found to be diploid with 2*n* = 2*x* = 14, which is a new diploid report in the southernmost geographic range of this polyploid complex.

## ﻿Introduction

The extended family of the Amaryllidaceae J. S. Hilaire, 1805, is one of the largest families of Asparagales. Among the subfamily Amaryllidoideae Burnett, 1835, species of tribe Narcisseae H.C. Lam et De Candolle, 1806, distributed in about 11 sections (Zonneveld 2008; Marques et al. 2017), constitute the most attractive group of plants due to their botanical characteristics, evolutionary trends, biochemical properties and ornamental interests. Despite the well-known phylogenetic relationships at the generic level (Santos-Gally et al. 2012; Marques et al. 2017), many questions remain still unclear at the specific level. This is probably due to the lack of unequivocal diagnostic characters, a likely consequence of a variation driven by a deeply reticulated evolutionary history with their high ability to hybridize (Rønsted et al. 2008; Aedo et al. 2013; García et al. 2014; López-Tirado 2018; González et al. 2019). Moreover, species of tribe Narcisseae, constitute an enigmatic model of karyotype evolution in terms of chromosome numbers, base number and origin of the polyploids. This is particularly true for species of genus *Narcissus* Linnaeus, 1753, which with about fifty species, exhibit a high variation in chromosome numbers ranging from 2*n* = 10 to 72 with occurrence of aneuploidy and polyploidy (Fernandes 1975; Brandham and Kirton 1987; Zonneveld 2008; Díaz Lifante et al. 2009; Sun et al. 2015). Many chromosome numbers have been reported and different basic numbers assumed but still unclarified. The most reported basic chromosome numbers in the literature were *x* = 5, *x* = 7, *x* = 10 and *x* = 11. In Algeria, species of genus *Narcissus* belong to three sections: Tazetteae De Candolle, 1806, Serotini Parlatore and Bulbocodii DC.

In the section Tazetteae, four species were recognized in the Algerian flora (Maire 1959). For this section, the common cited chromosome number was 2*n* = 2*x* = 20 (Fernandes 1975; Brandham and Kirton 1987) especially for *Narcissustazetta* Linnaeus, 1753, the most karyologically studied species. This species is widely distributed in the Mediterranean region, with the South Iberian Peninsula and Morocco as the center of diversity (Santos-Gally et al. 2012), and could reach the southern-west Asia, China and Japan (Hong 1982). These plants are characterized by a striking morphological variability expressed at the shape and color of corona and perianth divisions (Jones et al. 2008; Mifsud and Caruana 2010; Koopowitz et al. 2017). Comparison of the genome size by flow cytometry within *N.tazetta* had led Zonneveld (2008) to assume that this species is tetraploid with base number *x* = 5. In this same section, *Narcissuselegans* (Haworth) Spach, 1846, is also considered as tetraploid with 2*n* = 4*x* = 20 according to studies on genome size (Zonneveld 2008), *in situ* hybridization (Díaz Lifante et al. 2009) and phylogenetic analyzis (Marques et al. 2017). In section Serotini, the base number is also *x* = 5 and concerns *Narcissusserotinus* Linnaeus, 1753, sensu lato, in which three cytotypes have been observed: diploid (2*n* = 10), tetraploid (2*n* = 20) and hexaploid (2*n* = 30). These cytotypes were observed in populations respectively from the Iberian Peninsula and Morocco (Fernandes 1968; Aedo 2013), Sicily (Garbari et al. 1973; Phitos and Kamari 1974) and Central Italy (D’Amato 2004). The geographic range of the type *N.serotinus* would cover the Iberian Peninsula and northern Morocco. The presence of this taxon in Algeria, was recorded by all the botanists in XIX and XX centuries (Munby 1847; Battandier and Trabut 1895; Maire 1959; Quézel and Santa 1962) but remains doubtful and raises controversy as underlined in the Red List of IUCN (Juan Vicedo et al. 2018).

Although belonging to two different sections, *N.elegans* and *N.serotinus* would be involved as parents in the origin of natural hybrids such as *N.obsoletus* (Haworth) Steudel, 1841, and *N.miniatus* Donnison-Morgan, Koopowitz, Zonneveld, 2005, this latter species was discovered in Southern Spain (Donnison-Morgan et al. 2005). Both *N.miniatus* and *N.obsoletus* would be allohexaploid with 2*n* = 6*x* = 30 as highlighted by flow cytometry (Donnison-Morgan et al. 2005; Zonneveld 2008), and molecular cytogenetics (Díaz Lifante et al. 2009; Marques et al. 2010). In the district of Algiers, Quézel and Santa (1962) following Maire (1959), referred to a hybrid ×obsoletus (= N.elegansvar.intermedius J. Gay). Two other daffodils of the flora of Algeria, *N.pachybolbus* Durieu, 1847, and *N.papyraceus* Ker Gawler,1806, were often confused. Regarding their inflorescence and flowers, these species share many similarities with *N.tazetta*, that led Maire (1959) to consider them under N.tazettasubsp.pachybolbus (Durieu) Baker, 1888, and N.tazettasubsp.papyraceus (Ker Gawler) Baker, 1888. Yet, *N.pachybolbus* was discovered in 1846 by Durieu de Maisonneuve in the NW Algeria near Oran (Battandier and Trabut 1895), and was first considered as endemic to this region (Munby 1847; Battandier and Trabut 1895). *N.papyraceus* would be introduced from Europe, cultivated and then locally naturalized (Maire 1959). Phylogenetic analyses highlighted their very close relationships in the same clade (Jiménez et al. 2017; Marques et al. 2017) but were recognized today as distinct species by most nomenclatural databases.

Similar ambiguity arose in Algeria for *Narcissuscantabricus* De Candolle, 1815, of the section Bulbocodii. This species has been considered first under N.bulbocodiumsubsp.monophyllus (Durieu) Maire, 1931, then later, as a distinct species (Quézel and Santa 1962). *N.bulbocodium* is distinguished by a large polyploid series ranging from diploid 2*n* = 14 to octaploid 2*n* = 72 (Fernandes 1963, 1968; Zonneveld 2008; Marques et al. 2017) while *N.cantabricus* was known as diploid and tetraploid in Spain and Morocco.

Despite its central biogeographic position in the southwestern Mediterranean region, Algeria is characterized by an obvious lack of cytotaxonomic data leading to controversies about status and circumscription of many taxonomic units particularly within the Asparagales (Hamouche et al. 2010; Azizi et al. 2016; Khedim et al. 2016; Boubetra et al. 2017). Unfortunately, genus *Narcissus* is little known and poorly studied in our country.

The aim of this study is to fill the gap in the karyological data that links between the floras of the western Mediterranean region. It focuses on the main taxa of genus *Narcissus* recognized in the flora of Algeria, namely *N.tazetta*, *N.elegans*, *N.serotinus* sensu lato, *N.pachybolbus*, *N.papyraceus* and *N.cantabricus*. Chromosomal counting, structural parameters of the karyotype and the geographical distribution of the polyploidy have been done for each species. Karyological data were linked to morphological and chorological criteria in order to improve taxonomic and nomenclatural knowledge on the genus *Narcissus* in Algeria.

## ﻿Materials and methods

### ﻿Sampling and plant identification

**Figure 1.**
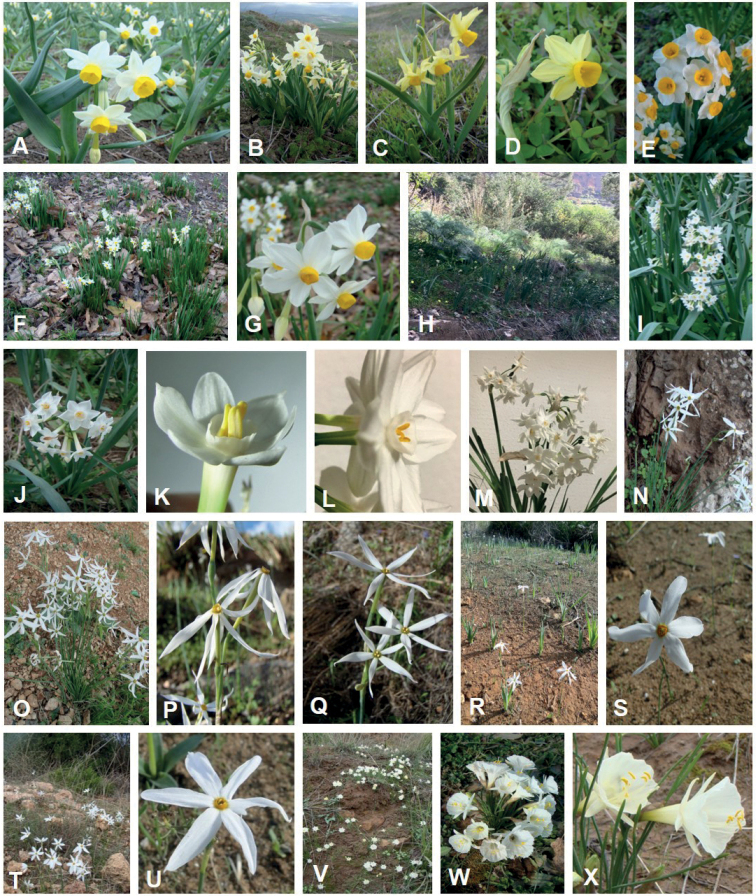
Habits and flowers of species of genus *Narcissus* from Algeria. *N.tazetta*: **A, B** Sidi Khélifa **C, D** Hammam Mélouane **E** Yakouren **F–G** Tizi Tghidet. N.pachybolbus: **H–K** El-Ourit. *N.papyraceus*: **L–M** Bologhine. *N.elegans*: **N–Q**. *N.serotinus*: **R–S** Ain Ftouh. *N.obsoletus*: **T–U** Sainte Salsa. *N.cantabricus*: **V–X** Mansourah. Photos by Rachid Amirouche.

Plant material used in this study consists of 32 natural populations of genus *Narcissus* sampled in contrasting ecological conditions along the east-west biogeographic gradient of the northern Algeria (Table 1). Systematic determinations were made using the main Algerian floras (Munby 1847; Battandier and Trabut 1895, 1902; Maire 1959; Quézel and Santa 1962) as well as floras from the Iberian Peninsula (Aedo 2013), from Morocco (Fennane et al. 2014), and from Tunisia (Le Floc’h et al. 2010). Status of the species and synonyms have been checked on the two main specialized websites, World Check List of Selected Plant Families (Govaerts 2015) and African plant database (Dobignard and Chatelain (2010–2013)). The studied taxa are presented in Table 2 and Fig. 1: *N.tazetta* and *N.elegans* are represented by several populations. Two natural populations of *N.pachybolbus* were narrowly located in the north-west of Algeria on the Mounts of Tlemcen, while those belonging to *N.papyraceus* are naturalized relics of cultivated plants. *N.serotinus* sensu lato is represented by populations collected over all the sampling area, some of which belong to *N.obsoletus. N.cantabricus* is narrowly located in the NW of Algeria at Tlemcen and near the Algerian-Moroccan border. From each site, 3–10 plants per taxon, with bulb, leaves and flowers, were collected. Voucher specimens were deposited at the Official Herbarium of ENSA (Algiers, Algeria) under numbers: ENSA13367-68 (*N.cantabricus*), ENSA13369-73 (*N.elegans*), ENSA13374-75 (*N.pachybolbus*), ENSA13376-77 (*N.papyraceus*), ENSA13378-81 (*N.serotinus*), ENSA13386-93 (*N.tazetta*).

**Table 1.** Coordinates, altitude and bioclimate of the collecting sites in northern Algeria.

Locality	Altitude (m)	Geographic coordinates	Bioclimate^†^	Collected species^‡^
Beni Bahdel	760	34°42'30.49"N, 01°31'08.33"W	Subhumid	*N.cantabricus*
Ain Ftouh	831	34°43'23.00"N, 01°27'13.00"W	Subhumid	*N.elegans / N.serotinus* s.l.*
Ahfir	1202	34°46'56.40"N, 01°24'54.70"W	Subhumid	*N.serotinus* s.l.
Mansourah	1160	34°50'12.60"N, 01°02'20.90"W	Subhumid	*N.cantabricus*
El-Ourit	739	34°25'00.00"N, 01°16'00.00"W	Subhumid	*N.pachybolbus*
Emir Abdelkader	460	35°13'34.50"N, 01°23'33.50"W	Subhumid	*N.pachybolbus*
Tessala	801	35°16'09.90"N, 00°46'16.80"W	Subhumid	*N.elegans*
Boutlélis	291	35°34'11.40"N, 00°54'00.00"W	Semi arid	*N.elegans / N.serotinus* s.l.
Santa Cruz	319	35°42'36.40"N, 00°39'51.10"W	Semi arid	*N.elegans*
Miliana	570	36°18'45.60"N, 02°16'22.06"E	Subhumid	*N.tazetta*
Mouzaïa	110	36°28'00.00"N, 02°41'00.00"E	Subhumid	*N.tazetta*
Chréa	1000	36°28'16.50"N, 02°55'37.40"E	Humid	*N.tazetta*
Chenoua	15	36°36'23.00"N, 02°22'21.00"E	Subhumid	*N.elegans*
Sainte Salsa	20	36°35'31.00"N, 02°26'58.00"E	Subhumid	*N.elegans / N.serotinus* s.l.
Hammam Mélouane	142	36°29'51.70"N, 03°03'29.60"E	Humid	*N.tazetta*
Ain Tagourait	219	36°35'00.00"N, 02°37'00.00"E	Subhumid	*N.elegans / N.serotinus* s.l.
Béni Messous	50	36°46'44.00"N, 02°58'30.10"E	Subhumid	*N.elegans*
Baraki	22	36°39'58.00"N, 03°05'30.00"E	Subhumid	*N.tazetta*
Baïnem	248	36°48'00.00"N, 02°58'00.00"E	Subhumid	*N.serotinus* s.l.
Bologhine	25	36°48'24.20"N, 03°02'24.50"E	Subhumid	*N.papyraceus*
El Alia	30	36°43'12.00"N, 03°10'00.00"E	Subhumid	*N.papyraceus*
Yakouren	700	36°43'49.90"N, 04°27'51.00"E	Humid	*N.tazetta*
Tizi Tghidet	750	36°44'48.00"N, 04°26'55.00"E	Humid	*N.tazetta*
Adekar	500	36°41'00.00"N, 04°40'00.00"E	Humid	*N.elegans*
Mont Gouraya	540	36°46'07.20"N, 04°49'50.00"E	Subhumid	*N.elegans*
El Aouana	74	36°46'00.00"N, 06°33'00.00"E	Humid	*N.elegans*
Aït Ali (Ziama)	970	36°37'04.40"N, 05°28'44.10"E	Humid	*N.serotinus* s.l.
Djebel Ouahch	983	36°24'24.50"N, 06°40'32.50"E	Subhumid	*N.tazetta*
Sidi Khélifa	864	36°21'08.90"N, 06°17'01.40"E	Subhumid	*N.tazetta*
Oued Djenane	302	36°49'17.10"N, 08°37'30.10"E	Humid	*N.tazetta*
El Aïoun	282	36°49'04.80"N, 08°37'29.40"E	Humid	*N.tazetta*
Tabarka (Tunisia)	80	36°52'21.70"N, 08°43'53.70"E	Humid	*N.tazetta*

**^†^** Bioclimate from Quézel and Santa (1962). **^‡^** Nomenclature from Maire (1959), Quézel and Santa (1962) and Dobignard and Chatelain (2010–2013). *N.serotinus sensu lato includes also *N.obsoletus*.

### ﻿Chromosome preparations

Chromosomal analysis was based on metaphase plates of root-tip cells from cultivated bulbs. Young roots (6–10 mm long) were pre-treated with 1% colchicine for 5–6 hours at room temperature, then fixed in ethanol-acetic acid (3:1) for 48 hours and conserved at 4 °C in ethanol 70°.The protocol was adapted from the Feulgen method (Jahier et al. 1992). The root-tips were hydrolysed in 1N hydrochloric acid for 7–12 min at 60 °C before stained with Schiff’s reagent in darkness for 1–2 hours. The squash was made in a drop of 45% acetic acid or carmine acetic. Metaphase plates were examined with a Zeiss Axiostar-Plus Microscope. Cells with good spreading of chromosomes were photographed.

**Table 2.** Comparison of the studied species of *Narcissus* based on the main diagnostic criteria.

Section	Tazetteae				Serotini	Bulbocodii
Species	*N.tazetta*	*N.pachybolbus*	*N.papyraceus*	*N.elegans*	*N.serotinus* sensu lato	*N.cantabricus*
Bulb length (mm)	28–58	39–77	37–62	15–38	13–22	19–21
Bulb width (mm)	15–58	37–68	30–55	12–34	7–20	10–15
Color of the tunic	black brown	black	black brown	black	black	black
Leaf number at flowering	2–8	3–5	3–6	1	0	1–5
Synanthous v*ersus* hysteranthous	synanthous	synanthous	synanthous	synanthous	hysteranthous	synanthous
Length of scape (mm)	80–510	204–496	370–672	102–523	85–240	104–137
Length of spathe (mm)	32–70	30–50	35–50	17–44	15–30	18–25
Number of flowers per scape	3–12	9–15	6–13	1–5	1 rarely 2	1
Hypanthial tube length (mm)	23–44	19–39	14–36	14–30	13–24	23–47
Hypanthial tube shape	cylindric	cylindric	cylindric	subcylindric narrow	subcylindric	obconic–funnel
Corona color	yellow–orange	white	white	olive yellow / greenish orange	variable yellow to orange	White rarely white–yellowish
Corona size	medium	medium	medium	small	small	very large
Color of tepals	white yellow	white	white	greenish white	greenish white	white
Pedicel length (mm)	18–52	19–40	27–62	9–40	11–25	3–4
Stamen position	emergent / not emergent	emergent	not emergent	not emergent	not emergent	emergent

**Note**: Diagnostic criteria from the main floras of Algeria: Battandier and Trabut (1902), Maire (1959), Quézel and Santa (1962).

### ﻿Karyotype analysis

Karyomorphometric measurements and the homologous chromosome ordering were made using the KaryoType Software 2.0 (Altınordu et al. 2016). Chromosomes are described according to the nomenclature of Levan et al. (1964) based on the arm ratio (r = long arm / short arm) and the centromeric index (CI% = short arm / long arm + short arm × 100): metacentric (m), submetacentric (sm), subtelocentric (st) and telocentric (t). Ideograms were drawn from at least 5 well-spread metaphase plates of different individuals. Karyotype asymmetry indices were estimated following the proposal of Peruzzi and Eroğlu (2013). The intrachromosomal asymmetry index is represented by the mean centromeric asymmetry MCA = A × 100, where A is the average ratio of long arm-short arm/long arm + short arm (Watanabe et al. 1999). The interchromosomal asymmetry index is the coefficient of variation of chromosome length CV_CL_ = A2 × 100 (Paszko, 2006) where A2 is the standard deviation of chromosome length/mean chromosome length (Romero Zarco 1986). The coefficient of variation of the centromeric index CV_CI_ = SCI / X̄ CI × 100 is the ratio between the standard deviation SCI and the mean centromeric index X̄ CI (Paszko 2006).

### ﻿Morphological analysis

In order to link karyological data to morphological relationships between the studied species, multivariate analyses were carried out using the diagnostic descriptors of vegetative and reproductive parts, some from personal observations (Table 3). Principal Component Analysis (PCA) were performed using the program R Software 4.1.0 (2021).

**Table 3.** List and abbreviations of the morphological characters used in the multivariate analysis.

**Quantitative characters**		**Shape of the scape**	
Bl	Bulb length (mm)	SScyl	cylindrical slightly ridged
Bw	Bulb width (mm)	SSang	angular ribbed
Ln	Leaf number	**Section of the scape**	
Scl	Scape length (mm)	SSfill	filled
Spl	Spathe length (mm)	SSfist	fistilous
Nf	Number of flowers by scape	**Shape of pedicel**	
Pl	Pedicel length (mm)	SPs	smooth
Hl	Hypanthial tube length (mm)	SPa	angular
Ns	Number of scape sheath/ scape	**Color of tunic bulb**	
Ow	Ovary width (mm)	TBcol1	black
Ol	Ovary length (mm)	TBcol2	brown
Tl	Outer Tepal length (mm)	**Color of corona**	
Tl/w	Ratio tepal length / width (mm)	Corcol1	orange bright
Tel	Tunic extension wrapping the scape (mm)	Corcol2	yellow-orange
Ch	Corona height (mm)	Corcol3	Yellow-lemon
		Corcol4	white
**Qualitative characters**		Corcol5	orange / orange greenish
**Leaves at flowering**		Corcol6	yellow / yellow greenish
Syn	Synanthous	**Shape of hypanthial tube**	
Hyst	Hysteranthous	Hysh1	subcylindric large
**Color of the tepals**		Hysh2	subcylindric narrow
Tc1	White	Hysh3	cylindric
Tc2	yellow	Hysh4	obconic funnel

## ﻿Results

**Figure 2.**
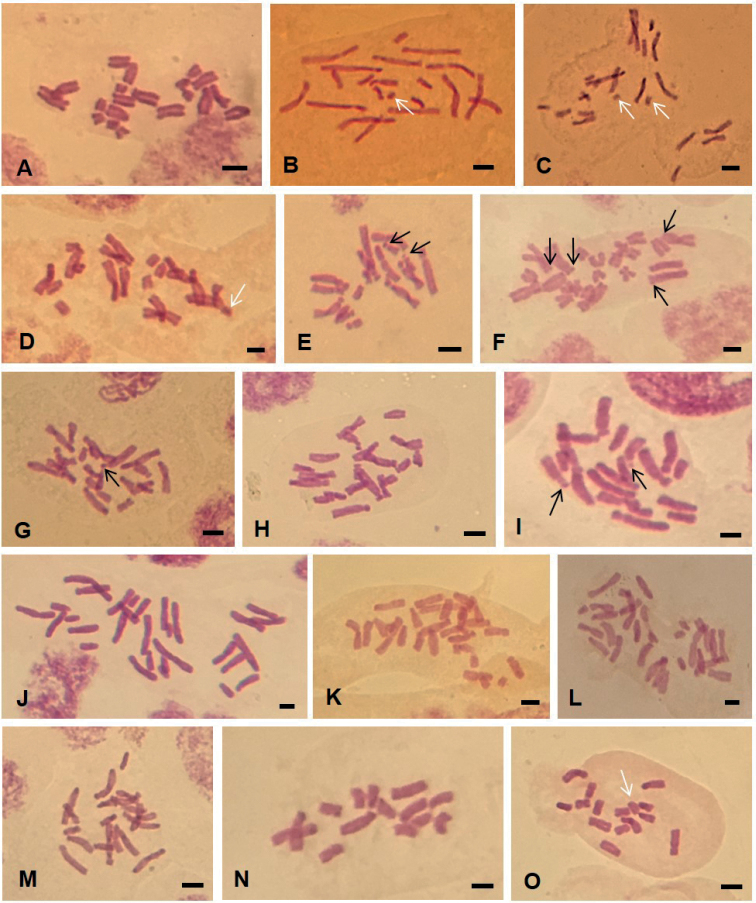
Somatic metaphases of some species of genus *Narcissus* from Algeria. **A–D***N.tazetta*: **A** 2*n* = 20 El Aïoun **B** 2*n* = 20 + 1 Sidi Khélifa **C** 2*n* = 20 + 2 Sidi Khélifa **D** 2*n* = 20 + 1 Oued Djenane E 2*n* = 20 Tabarka **F***N.pachybolbus* 2*n* = 22 Emir Abdelkader **G***N.papyraceus* 2*n* = 22 Bologhine **H–I***N.elegans*: **H** 2*n* = 20 Sainte Salsa **I** 2*n* = 20 Ain Tagourait **J–M***N.serotinus* s.l. : **J–K** 2*n* = 30 Ain Tagourait, Sainte Salsa **L** 2*n* = 28 Ain Ftouh **M** 2*n* = 20 Aït Ali. **N–O***N.cantabricus*: **N** 2*n* = 14 **O** 2*n* = 14 + 1 Mansourah. Black arrows indicate satellites. White arrows indicate supernumerary chromosomes. Scale bar: 10 μm.

**Figure 3.**
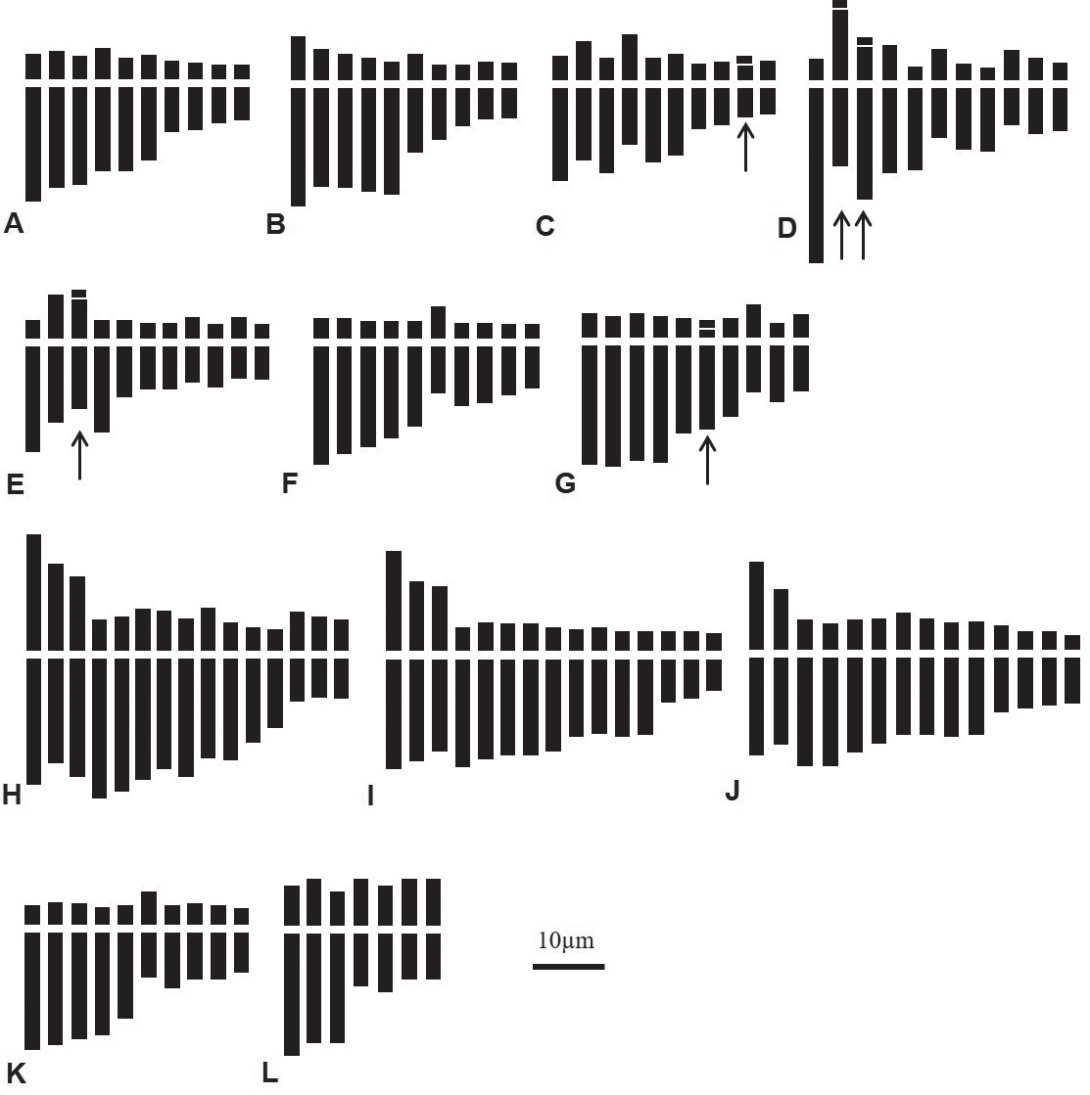
Ideograms of the studied species of genus *Narcissus* in Algeria **A***N.tazetta* 2*x* (El Aïoun, Yakouren, Hammam Mélouane, Baraki, Sidi Khélifa, Mouzaïa) **B***N.tazetta* 2*x* Oued Djenane **C***N.tazetta* 2*x* Tabarka **D***N.pachybolbus* (Emir Abdelkader, El Ourit) 2*x.***E***N.papyraceus* (Bologhine, El Alia) **F***N.elegans* 2*x* (Boutlélis, Tessala, Béni Messous, Chenoua, Sainte Salsa, Santa Cruz) **G***N.elegans* 2*x* Ain Tagourait **H***N.serotinus* s.l. 6*x* Ain Tagourait **I***N.serotinus* s.l. 6*x* Sainte Salsa **J***N.serotinus* s.l. 2*n* = 28 Ain Ftouh **K***N.serotinus* s.l. 4*x.* Aït Ali **L***N.cantabricus* 2*x* Mansourah. Arrows indicate satellites. Scale bar: 10 µm.

Chromosome numbers, ploidy level and characteristics of the karyotypes of the examined populations are summarized in Table 4. Comparisons of chromosome numbers from this study with those reported in the literature are summarized in Table 5. Representative metaphases and ideograms are shown in Figs 2, 3 respectively. Following the karyological data, we carried out morphological analysis for the studied taxa i.e., *N.tazetta*, *N.elegans*, *N.pachybolbus*, *N.papyraceus*, *N.serotinus* and *N.cantabricus*. Morphological analyses aim to highlight on interspecific variability in relation to karyological characteristics of the species.

**Table 4.** Chromosome number, ploidy level and karyotype characteristics of the examined populations of genus *Narcissus* in Algeria.

Species/ Populations	Ind/ cells	*x*	*2n*	Pl	Karyotype formula	THL	Asymmetry indices					
							Stebbins	A1	A2	MCA	CV_CL_	CV_CI_
***Narcissustazetta* L.**												
Tabarka (Tunisia)	4/16	10	20	2x	4m + 10sm (2sat) + 6st	114.75	3B	0.54	0.36	38.85	35.83	27.56
Oued Djenane	5/20	10	20	2x	2m + 8sm + 10st	126.12	3B	0.62	0.40	46.68	39.93	29.23
Sidi Khélifa	3/8	10	20	2x	10sm + 10st	126.17	3B	0.66	0.38	50.27	38.45	20.54
El Aïoun	4/12											
Hammam Mélouane	7/31											
Yakouren	3/15											
Baraki	5/38											
Mouzaïa	5/15											
***Narcissuspachybolbus* Dur.**												
Emir Abdelkader	5/21	11	22	2x	6m (2sat) + 6sm (2sat) + 8st + 2t	151.92	3B	0.53	0.43	40.18	43.06	40.73
El-Ourit	3/10											
***Narcissuspapyraceus* Ker Gawl.**												
Bologhine	6/36	11	22	2x	6m (2sat) + 12sm + 4st	115.50	3B	0.55	0.38	39.86	37.62	29.57
El Alia	3/10											
***Narcissuselegans* (Haw.) Spach**												
Ain Tagourait	4/8	10	20	2x	2m + 2sm + 14st + 2t (2sat)	145.23	3B	0.72	0.29	58.73	29.00	46.78
Boutlélis	3/14	10	20	2x	2m + 4sm + 14st	125.32	2B	0.69	0.32	54.15	31.94	32.92
Santa Cruz	4/9											
Sainte Salsa	7/28											
Béni Messous	3/9											
Chenoua	3/20											
Tessala	4/8											
***Narcissusserotinus* L.**												
Aït Ali	2/30	5	20	4x	2m + 6sm + 12st	66.01	3B	0.69	0.33	55.29	34.40	39.40
Ain Ftouh	6/10	-	28	-	-	-	-	-	-	-	-	-
Boutlélis	4/10											
Sainte Salsa	6/10	5	30	6x	6m + 6sm + 18st	78.89	3C	0.58	0.39	43.53	39.34	35.20
Ain Tagourait	4/12	5	30	6x	1M + 11m + 6sm + 12st	101.89	3B	0.47	0.37	34.86	37.15	38.07
***N.obsoletus* (Haw.) Steud**												
Ain Ftouh	4/10	-	30	-	-	-	-	-	-	-	-	-
Boutlélis	5/15											
Sainte Salsa	3/10											
***Narcissuscantabricus* DC.**												
Mansourah	5/15	7	14	2x	6m + 4sm + 4st	67.80	3A	0.45	0.27	31.33	26.91	29.16

**Abbreviations: Ind/cells** numbers of individuals/metaphase plates used for ideogram construction, **Pl** ploidy, **THL** Total Haploid Length, m, sm, st, t: type of chromosome according to Levan et al. (1964), sat: satellite, **MCA** Mean Centromeric Asymmetry, **CV_CL_** Coefficient of Variation of Chromosome Length, **CV_CI_** Coefficient of Variation of Centromeric Index, **A1**, **A2** intra and inter chromosomal asymmetry index, **Stebbins** Karyotype asymmetry degree.

### *Narcissustazetta* Linnaeus, 1753, sensu lato

≡ Narcissustazettasubsp.tazetta

This species has many heterotypic synonyms. It is widespread in the north of Algeria and shows a high polymorphism with regard to the color of the perianth and corona (Fig. 1). The somatic chromosome number is generally 2*n* = 20 (Fig. 2A) and constant in all the karyologically examined populations (Table 4). Sometimes 1 to 2 supernumerary chromosomes have been observed such as in populations of Sidi Khélifa (Fig. 2B, C), and Oued Djenane (Fig. 2D). Three different cytotypes were observed (Table 3). The karyotypic formula 10sm + 10st was found in most of populations. Two other cytotypes concern populations located towards the east, Oued Djenane and Tabarka, with 2m + 8sm + 10st and 4m + 10sm (2sat) + 6st, respectively. The last two karyotypes are distinguished by a lower asymmetry indices MCA, 38.85 and 46.68 respectively, *versus* 50.27 for the remain populations. Satellites were observed in population of Tabarka only (Fig. 2E), which is characterized by a relative smaller total haploid length (THL = 114.75 μm).

**Table 5.** Chromosome numbers of the studied species of genus *Narcissus* from Algeria compared to reports from the literature.

Species	This study	Reports from the literature	
*N.tazetta* L.	2*n* = 20 2*n* = 20 + 1	2*n* = 14, 20, 22, 24, 28, 30, 32	Sharma and Sharma (1961), Brandham and Kirton (1987)
		2*n* = 20	Hong (1982), Garbari et al. (1988), Baldini (1990), Dominicis et al. (2002), Aquaro et al. (2007), Zonneveld (2008), Díaz Lifante et al. (2009), Marques et al. (2010), Boukhenane et al. (2015)
		2*n* = 10, 20, 21, 22, 30, 31, 32	Aedo (2013)
*N.pachybolbus* Dur.	2*n* = 22	2*n* = 22	Maugini (1953), Brandham and Kirton (1987)
		2*n* = 36	Aedo (2013)
*N.papyraceus* Ker Gawl.	2*n* = 22	2*n* = 22	Brandham (1942), D’Amato (2004), Aedo (2013), Samaropoulou et al. (2013), Marques et al. (2017)
*N.elegans* (Haw.) Spach	2*n* = 20	2*n* = 20	Fernandes (1966), Brandham and Kirton 1987, D’Amato (2004), Donnison-Morgan et al. (2005), Zonneveld (2008), Díaz Lifante et al. (2009), Marques et al. (2012), Aedo (2013), Troia et al. (2013)
		2*n* = 30	Brandham and Kirton (1987)
*N.serotinus* L.	2*n* = 20 2*n* = 28 2*n* = 30	2*n* = 10	Fernandes (1968, 1975), Brandham and Kirton (1987), Zonneveld (2008)
		2*n* = 10 (15)	Aedo (2013)
		2*n* = 20	Garbari et al. (1973), Phitos and Kamari (1974)
		2*n* = 30	D’Amato (2004), Zonneveld (2008)
*N.obsoletus* (Haw.) Steud.	2*n* = 30	2*n* = 30 (20, 29, 31, 45)	Aedo 2013
		2*n* = 30	Díaz Lifante et al. (2009), Troia et al. (2013)
*N.cantabricus* DC.	2*n* = 14 2*n* = 14 + 1	2*n* = 14	Zonneveld (2008), Aedo (2013)
		2*n* = 28	Zonneveld (2008)

### *Narcissuspachybolbus* Durieu, 1847

≡ Narcissustazettasubsp.pachybolbus (Durieu) Baker, 1888

≡ Narcissuspapyraceussubsp.pachybolbus (Durieu) D.A. Webb, 1978

*Narcissuspachybolbus* is narrowly distributed in NW Algeria mainly in the region of Tlemcen. Two populations were sampled at Emir Abdelkader and El Ourit. Both are diploids with 2*n* = 2*x* = 22 and share the same karyotype formula 6m (2sat) + 6sm (2sat) + 8st + 2t (Table 3). This species has the highest total haploid length THL = 151.92 µm. The karyotype is distinguished by terminal satellites on the second and third largest submetacentric and subtelocentric pairs (Figs 2F, 3D).

### *Narcissuspapyraceus* Ker Gawler, 1806

≡ Narcissustazettasubsp.papyraceus (Ker Gawler) Baker, 1888

This species has long been confused with the spontaneous *N.pachybolbus* due to strong similarities in the flower. *N.papyraceus* is an ancient cultivated species locally naturalized in Algeria. Two populations were found in the cemeteries of Algiers at Bologhine (ex. Saint Eugène) (Fig. 2G) and El Alia. Both populations show 2*n* = 2*x* = 22 chromosomes with the same karyotype formula 6m (2sat) + 12sm + 4st (Table 3). The karyotype of this species differs from that of *N.pachybolbus* by the presence of satellites on the 3^rd^ metacentric pair. For this taxon, the coefficients of variation of the length of the chromosomes (CV_CL_ = 37.62) as well as the centromeric index (CV_CI_ = 29.57) are lower. Despite their morphological similarity, the THL of *N.papyraceus* is closer to that of *N.tazetta* than that of *N.pachybolbus* (Table 3, Fig. 3E).

### *N.elegans* (Haworth) Spach, 1846

≡ *Hermioneelegans* Haworth, 1831

*N.elegans* is encountered mainly in the Tell of the biogeographical sectors of Oranie, Algiers and the Kabylies. Seven representative populations were karyologically examined. The same diploid somatic chromosome number 2*n* = 20 are observed in all the samples with *x* = 10 (Table 4). However, two slightly different karyotypes were observed (Table 4, Fig. 2H, I). The most frequent concerns populations from the western region (Boutlélis, Santa Cruz, Tessala) and the center region (Chenoua, Sainte Salsa, Béni Messous) (Fig. 2H). The karyotype formula is 2m + 4sm + 14st. The second karyotype with formula 2m + 2sm + 14st + 2t (2sat) was observed only in the population of Ain Tagourait (Fig. 2J). It is distinguished by a coefficient of variation of centromeric index CV_CI_ (46.78 *vs* 32.92) and total haploid length THL (145.23 µm *vs* 125.32 µm).

### *N.serotinus* Linnaeus, 1753, sensu lato

= Narcissusserotinusvar.emarginatus Chabert, 1889

Including *N.obsoletus* (Haworth) Steudel, 1841

≡ *Hermioneobsoleta* Haworth, 1819

*N.serotinus* sensu lato is found in the same biogeographical areas than *N.elegans*, however with a much smaller occurrence. Sometimes, the two species grow in sympatry as in Ain Ftouh, Boutlélis, Ain Tagourait and Sainte Salsa. Five populations belonging to *N.serotinus* s.l. were examined and three chromosome numbers were observed, 2*n* = 20, 2*n* = 28 and 2*n* = 30 (Table 4). Most of the individuals of these populations from the central region, share the same chromosome number 2*n* = 30 corresponding to hexaploid level with base number *x* = 5. The karyotype formulas were slightly different particularly for THL and asymmetry indices A1 and MCA (Table 4, Fig. 2J, K, Fig. 3H, I). The cytotypes with 2*n* = 28 are unusual and concern individuals of two populations from the far west at Ain Ftouh and Boutlélis (Fig. 2L, Fig. 3J). The chromosome number 2*n* = 20 is observed for one population only of Aït Ali located toward east of the sampling area (Table 4, Fig. 2M, Fig. 3K). This tetraploid karyotype is moderately asymmetric and distinguished by a small total haploid length (THL = 66.01 µm).

### *Narcissuscantabricus* De Candolle, 1815

= Narcissusbulbocodiumsubsp.monophyllus (Durieu) Maire, 1931

For this baetico-rifan species, two populations were sampled in NW Algeria, on clayey-marly slope in Mansourah forest near Tlemcen and on the edge of Lake Beni Bahdel towards the Algerian-Moroccan border. A diploid chromosome number was established 2*n* = 2*x* = 14 (Table 3, Fig. 2N, Fig. 3L). The karyotypic formula is 6m + 4sm + 4st with respectively intra and inter chromosomal asymmetry indices, A1 = 0.45 and A2 = 0.27 (Table 4). The total haploid length THL is 67.80 μm. One supernumerary chromosome was sometimes observed 2*n* = 14 + 1 (Fig. 2O).

## ﻿Discussion

**Figure 4.**
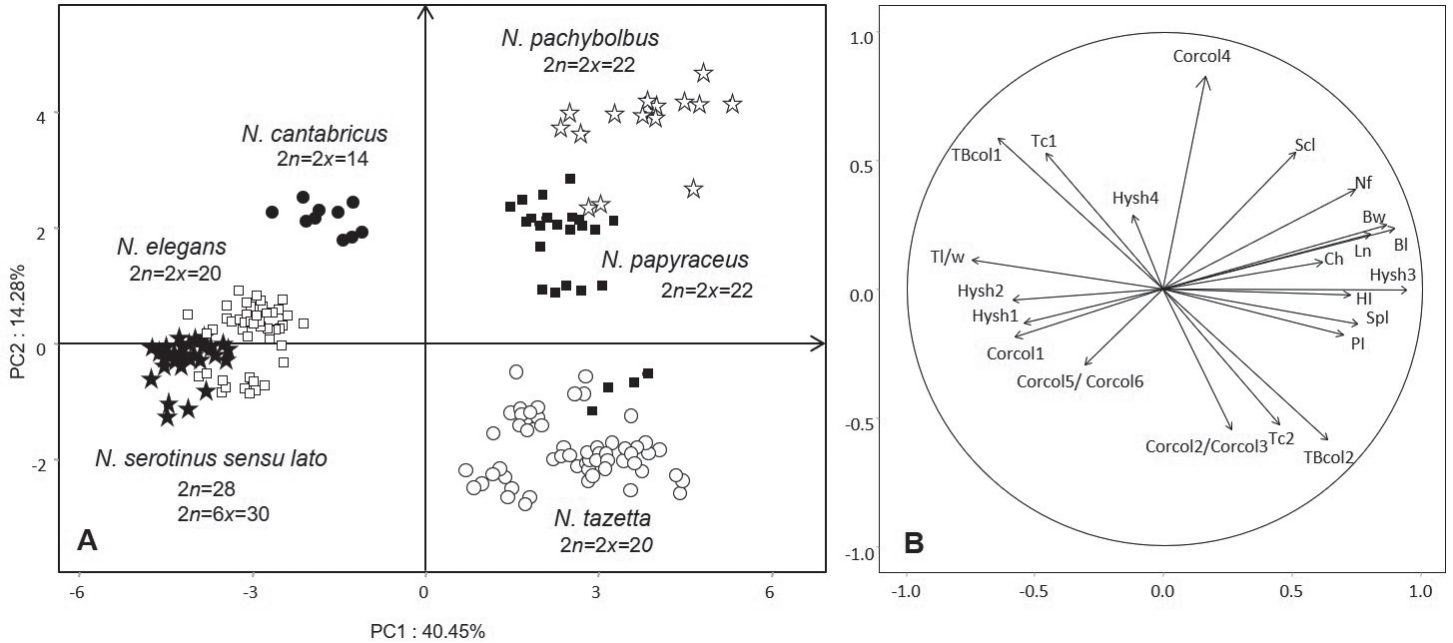
Principal Component Analysis of the main taxa of genus *Narcissus* in Algeria **A** overall scatter plot of 186 individuals representative of all the studied species **B** loading of the 24 quantitative and qualitative morphological and floral traits on the circle of correlations (see Table 3 for abbreviations). The distribution on PC1 and PC2 underlines the grouping of individuals belonging to *N.tazetta*, *N.pachybolbus* and *N.papyraceus* in opposition to *N.serotinus* sensu lato, *N.elegans* and *N.cantabricus*. The main discriminating criteria are relative to the length of the scape (Scl) and size of the bulb (Bl, Bw) as well as the number of flowers per inflorescence (Nf) and especially the height (Ch) and color of the corona (Corcol). This analysis highlights the strong relationships between the serotinus sensu lato type with the *elegans* type, likewise for *N.papyraceus* and *N.pachybolbus*.

In order to link karyological and morphological data of the Algerian species, Principal Components Analysis (PCA) were performed on the basis of the main taxonomic criteria (see Table 3). Figure 4 underline strong interspecific differentiation between the studied taxa. Compared to PC1, the *N.tazetta-pachybolbus*-*papyraceus* species constitute a group clearly opposed to *N.cantabricus*, *N.serotinus* s.l. and *N.elegans*. The last two species *N.serotinus* s.l. and *N.elegans* show morphological affinities. This distribution is in full correlation with the chromosome numbers.

### ﻿The *N.tazetta-pachybolbus-papyraceus* group

All of the ten Algerian populations belonging to *N.tazetta* share the same chromosome number 2*n* = 20 with sometimes one or two B chromosomes. This somatic number was previously reported by Boukhenane et al. (2015) in the district of Constantine. This number is the most commonly observed in the Mediterranean region such as in Greece, Cyprus, Italy, and Southern France (Hong 1982; Garbari et al. 1988; Baldini 1990; Dominicis et al. 2002; Aquaro et al. 2007). Other chromosome numbers have been reported e.g., 2*n* = 14, 20, 22, 24, 28, 30 and 32 (Sharma and Sharma 1961; Brandham and Kirton 1987). The occurrence of one or two B chromosomes makes uncertain the base number (Baldini 1995; Dominicis et al. 2002; Zonneveld 2008). Indeed, most of the studies mention only the somatic chromosomal numbers (2*n*) without indication on the base number. Hong (1982) refer to *x* = 10 following the pioneering work of Fernandes (1951, 1966) who had already suggested three base numbers *x* = 7, *x* = 10 and *x* = 11 withing genus *Narcissus*. While, Brandham and Kirton (1987) have assumed a tetraploid (2*n* = 4*x* = 20) and hexaploid (2*n* = 6*x* = 30) levels for *N.tazetta*. On the basis of an exhaustive study on genome size measured by flow cytometry, Zonneveld (2008) has also assumed *x* = 5 as common base number for *N.tazetta*, *N.elegans* and *N.serotinus*. Most of the Algerian populations of *N.tazetta* show karyotypes expressing roughly similar formula. However, two populations collected in the eastern part near the Tunisian border (Oued Djenane, Tabarka), are distinguished by a less asymmetric karyotype. That of Tabarka, in Tunisia, was singularized by satellites on the 9^th^ submetacentric chromosome pairs contrary to those observed on the 6^th^ and 7^th^ subtelocentric chromosome pairs for some *tazetta* taxonomic units (Maugini1953; Hong 1982; Dominicis et al. 2002; Boukhenane et al. 2015).

Due to their morphological similarities, Maire (1959) had considered *N.pachybolbus* and *N.papyraceus* as subspecies of *N.tazetta*. Although *N.papyraceus* has never been reported in the ancient flora of Algeria (Munby 1847; Battandier and Trabut 1895, 1902). *N.pachybolbus* first described in NW of Algeria by Durieu (1846), is currently considered as an Ibero-Mauritanian species quoted in Morocco (Fennane et al. 2014) and Spain (Aedo 2010). For the Algerian populations of *N.pachybolbus* we have counted a diploid number of 2*n* = 2*x* = 22 consistent with previous studies (Maugini 1953; Brandham and Kirton 1987). However, in Flora Iberica, Aedo (2013) mentions 2n = 36. These two different chromosome numbers in two distinct territories suggest the need for a revision of this taxon. In our knowledge, the karyotypic formula is here provided for the first time: 6m (2sat) + 6sm (2sat) + 8st + 2t. A few karyological studies were devoted to this species. Brandham and Kirton (1987) have described just talk about a karyotype significantly different consisting of “…8 large acrocentric and 14 smaller acrocentric or submetacentric chromosomes”. Our samples of *N.papyraceus* exhibit also 2*n* = 22 chromosomes confirming previous reports (D’Amato 2004; Aedo 2013; Samaropoulou et al. 2013; Marques et al. 2017). The structure of the karyotype of *N.papyraceus* has been widely discussed by Brandham and Kirton (1987) and D’Amato (2004). Satellites have been observed on the 6^th^ and 7^th^ chromosomes pairs in contrast to Algerian samples which exhibit satellites on the 3^rd^ pair only. Although the karyotypic structures of these two species were considered as similar by Brandham and Kirton (1987), the Algerian samples of *N.pachybolbus* and *N.papyraceus* differ notably in the asymmetry indices. Contrary to the karyological diversity observed between *N.pachybolbus* and *N.papyraceus*, trees resulting from molecular phylogenies reconstruction show a polytomy indicating a very close relationship between these two species (Santos-Gally et al. 2012; Marques et al. 2017).

Morphologically *N.tazetta*, *N.pachybolbus* and *N.papyraceus* constitute three distinct clusters (Fig. 4). In respect to PC2, *N.pachybolbus* and *N.papyraceus* (2*n* = 22) are clearly in opposition to *N.tazetta* (2*n* = 20). The main morphological characters involved in this differentiation, relate to the color of the corona, the size and color of the outer layers of the bulb as well as the number of flowers per scape. Although sharing the same chromosome number 2*n* = 22, *N.pachybolbus* differs from *N.papyraceus* by higher values in the size of the bulb, the number of flowers per scape and emerging stamens from the corona (Fig. 1, Table 2). *N.papyraceus* is in intermediate position between *N.pachybolbus* and *N.tazetta*. The latter shows a high morphological variability expressed by small to medium bulb with rather brown outer tunics, a perianth white to yellow and a corona lemon to orange. These results agree with molecular phylogenies (Santos-Gally et al. 2012). The specific statute of *N.pachybolbus* and *N.papyraceus* agree with recent typification and taxonomic updating on daffodils (Aedo 2010; Koopowitz et al. 2017).

### ﻿*Narcissuselegans*, *N.serotinus* and *N.obsoletus*

**Figure 5.**
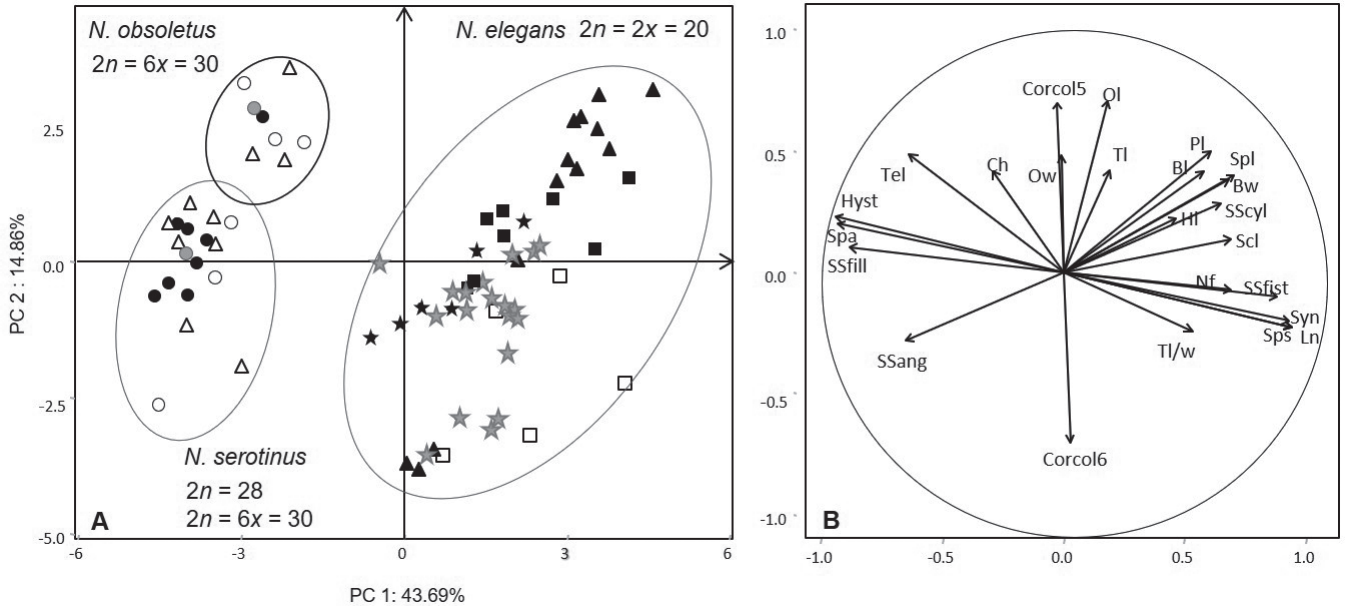
Principal components analysis focused on populations of *Narcissuselegans* and *N.serotinus* sensu lato **A** scatter plot on the first two PC of individuals of each taxon **B** loading of the morphological variables on the circle of correlations (see Table 3 for abbreviations). Morphologically *N.elegans* is well separated from *N.serotinus* sensu lato, by its synanthous habit (Syn), the number of flowers per scape (Nf), a full section of the scape (SSfill). With respect to PC2, individuals of *N.serotinus* s.l. are distributed in two opposed groups by the color of the corona. In the negative side individuals with yellow corona (Corcol6) correspond to *N.serotinus* type. Others individuals with orange corona (Corcol5) belong to *N.obsoletus* type. *N.serotinus* s.l.: black circle - St Salsa, white circle - Ahfir, white triangle - Ain Ftouh, gray circle - Boutlélis. *N.elegans*: gray star - Boutlélis, white square - Ain Tagourait, black triangle - Santa Cruz, black square - Chenoua, black star - St Salsa.

*Narcissuselegans* and *N.serotinus* s.l. have been described in all ancient floras of Algeria (Desfontaines 1798; Munby 1847; Battandier and Trabut 1895, 1902; Maire 1959; Quézel and Santa 1962) and several intermediate forms and putative hybrids have been reported. In Zonneveld (2008) and Marques et al. (2017), these two taxa were placed in section Serotini and section Tazettae, respectively. Some authors have grouped them together in the section Tazetteae (Santos-Gally et al. 2012). Regarding the Algerian material, these two species show close morphological relationships (Fig. 5). *N.serotinus* sensu lato within the meaning of Maire (1959) and Quézel and Santa (1962), is distinguished from *N.elegans* by its hysteranthous and smaller habit, and by “stable” characters such as single, or rarely 2, flowers per scape, larger and obtuse outer tepals. The other diagnostic descriptors, in particular the color and the shape of the corona, are variable and therefore difficult to use in practice. The inconstancy of these characters was noted by Maire (1959) and Quézel and Santa (1962) who had described around Algiers, intermediate forms attributed to × *N.obsoletus* (= *Hermioneobsoleta*), as a putative hybrid *N.elegans × serotinus*. These two species are also distinguished by their karyological characteristics. The natural hybrid × *N.obsoletus* was underlined by DNA content of specimens from Spain and Morocco (Donnison-Morgan et al. 2005).

In our study, *N.elegans* has a constant somatic chromosome number 2*n* = 20 reported also in the literature but often without mention of the base number (D’Amato 2004; Díaz Lifante et al. 2009; Aedo 2013; Troia et al. 2013). The reconstructed ideograms of *N.elegans* show groupings preferentially in pairs of homologous suggesting a diploid level with *x* = 10. This is inconsistent with Donnison-Morgan et al. (2005), Zonneveld (2008) and Marques et al. (2012) who have assumed that *N.elegans* is tetraploid with *x* = 5. The karyotypic structure of *N.elegans* compared to that of *N.tazetta* from Algeria, shows similarities in agreement with the first assumptions of Fernandes (1966). The values of THL and the asymmetry indices of these two species vary within the same interval, except for CV_CI_ and CV_CL_ which are different. These differences would be due to chromosome structural changes as suggested by D’Amato (2004).

The Algerian populations belonging to *N.serotinus* sensu lato, display three somatic chromosome numbers 2*n* = 20, 2*n* = 28 and 2*n* = 30. The karyotype formula and the ideograms let suppose a base number *x* = 5 and consequently tetraploid and hexaploid levels. The tetraploids (2*n* = 20) were encountered in Sicily (Garbari et al. 1973) and in Greece (Phitos and Kamari 1974), the hexaploids (2*n* = 30) were quoted in Italy (D’Amato 2004; Troia et al. 2013). Diploid forms 2*n* = 2*x* = 10 were mentioned in Iberian Peninsula and Morocco by Fernandes (1968, 1975), Brandham and Kirton (1987) and Aedo (2013). This diploid cytotype (2*n* = 10) is considered very rare and would represent the *N.serotinus* type narrowly distributed in this region (Zonneveld 2008). In the literature, the most accepted and widespread ploidy level for *N.serotinus* remains the tetraploid 2*n* = 20. The hexaploid would raise controversy over its systematic statute. Analysis of genome size by flow cytometry led Zonneveld (2008) to attribute the hexaploid cytotype to *N.miniatus* which would be also confused with *N.serotinus*. Subsequent studies (Díaz Lifante et al. 2009; Marques et al. 2010, 2012, 2017) support that *N.miniatus* is an allohexaploid from *N.serotinus* (2*n* = 10) × *N.elegans* (2*n* = 20). This hexaploid form, firstly located in Spain, have a geographic range through the northern Mediterranean edge from Italy toward Lebanon, Palestine until Syria (Zonneveld 2008). On the contrary, the hexaploid specimens found by Troia et al. (2013) in Mazara del vallo (Sicily, Italy) have been attributed to *N.obsoletus*, which would have a larger geographic distribution area, especially in North Africa. Díaz Lifante et al. (2009) confirmed that the hexaploid cytotype of Spain and Greece belong to *N.obsoletus*. In our study, the karyologically examined populations are all mixed and would include individuals belonging to *N.serotinus* and *N.obsoletus*. The PCA focused on specimens of *N.serotinus* sensu lato and *N.elegans* (Fig. 5) show that the cytotypes with 30 and 28 chromosomes are all distributed along PC2. This distribution is determined by the color of the corona. All individuals located in positive pole of PC2, have orange corona and would correspond to *N.obsoletus*. At the opposite, individuals with yellow corona correspond to *N.serotinus*. This differentiation is consistent with the observations of Díaz Lifante and Andrès Camacho (2007) and Koopowitz et al. 2017. In Algeria, *N.obsoletus* was often misidentified and sometimes confused with *N.serotinus*. In our opinion, the two species *N.serotinus* (4*x*, 6*x*) and *N.obsoletus* (6*x*) are well present in Algeria in mixed populations. The hexaploid cytotypes are located mainly in the center region near Algiers (Ain Tagourait, Sainte Salsa). The unusual cytotypes 2*n* = 28 were encountered in the northwest near Oran (Boutlélis) and Tlemcen (Ain Ftouh), could be due to aneuploidy event (Figs 4, 5). The tetraploid cytotypes (2*n* = 20) belongs to *N.serotinus* are rare in Algeria and its encountered rather in pure populations, localized mostly in the eastern region. In the IUCN Red List of Threatened Species, *N.serotinus* was considered uncertain in our country (Juan Vicedo et al. 2018).

### ﻿Narcissuscantabricus

The presence of *N.cantabricus* in Algeria, was subject to controversy with *N.bulbocodium*. *N.cantabricus* was not mentioned previously in the floras of North Africa. Maire (1959) had described this species under N.bulbocodiumsubsp.monophyllusvar.typicus with *Corbulariamonophylla* as synonym. *C.monophyla* was initially reported in Algeria by Battandier and Trabut (1895) and then considered as synonym of *N.monophyllus* before being accepted by Quézel and Santa (1962) under *N.cantabricus*. *N.cantabricus* is distinguished from *N.bulbocodium* by a “white or slightly yellowish flower” (Battandier and Trabut 1895). These two species are mentioned in *Flora Iberica* (Aedo 2013) and *Flore Pratique du Maroc* (Fennane et al. 2014). Phylogenetic analyzes carried out successively by Fonseca et al. (2016) and Marques et al. (2017) confirmed their separation. The Algerian populations of *N.cantabricus* is diploid (2*n* = 14) with sometimes one B chromosome. The karyotype established here for the first time for this species, is rather symmetrical comprising mostly meta and submetacentric chromosomes. In the literature, diploid cytotypes were reported on the Cantabrian Mounts in the north, and in the center of Spain, while tetraploids are quite rare and found in Morocco on the Anti-Atlas (Zonneveld, 2008). Therefore, the Algerian diploids would be the southernmost within the geographic range of this species. Although the haploid amount of DNA is similar in the two species, it seems that *N.cantabricus* derived from *N.bulbocodium* following structural changes (Zonneveld, 2008). *N.bulbocodium* is distinguished by a high polyploid series from 2*x* to 8*x* with 2*n* = 72 as the highest chromosome number (Fernandes 1963; Fernandes and Franca 1974; Brandham and Kirton 1987; Marques et al. 2017). *N.bulbocodium* is an Ibero-Mauritanian whose polyploids propagate from North to South towards Morocco and from West to East through the Maghreb as already hypothesized by Fernandes (1951). This geographical distribution of the polyploidy is similar for the two species, and therefore the Algerian diploids of *N.cantabricus* constitute original and interesting material. The supernumerary chromosomes in the Algerian peripheral diploids, would express an adaptive response to aridity.

## ﻿Conclusion

Overall, this work has contributed with new information supplementing our knowledge on chromosome numbers, karyotypes and ploidy levels of species of the genus *Narcissus*. The relationships between karyological and morphological characteristics made it possible to confirm and/or update the nomenclature and the taxonomy of species of genus *Narcissus* in Algeria. Therefore, seven main taxa have been recognized. Into the section Tazetteae, *N.tazetta* and *N.elegans* are diploids showing 2*n* = 2*x* = 20, while *N.pachybolbus* and *N.papyraceus* have 2*n* = 2*x* = 22 chromosomes. Section Serotini is represented by both tetraploid and hexaploid *N.serotinus* (2*n* = 20, 2*n* = 30) and also by the hexaploid *N.obsoletus* (2*n* = 30). These two species are very similar morphologically and have long been confused with each other in the field. Among *N.serotinus* type, tetraploids are rare comparatively to hexaploids. The distribution of *N.obsoletus* (6*x*) is widespread from west to east through various habitats. *N.cantabricus* show 2*n* = 2*x* = 14 and one recurrent B chromosome and constitute the southernmost diploids, providing new element for our understanding of the distribution of polyploidy within this species.
